# Supplementary material for: Ciliary Hedgehog signaling regulates cell survival to build the facial midline
Source: eLife. 2021 Oct 21;10:e68558. doi: 10.7554/eLife.68558 (PMC8592574; doi:10.7554/eLife.68558)
Supplement: Supplementary file 1. — File contains all sequencing primer pairs used for genotyping of all mouse strains used in this study. In addition, file contains primer sets used for RT-qPCR experiments. Inquiries regarding primers should be sent to Jeremy Reiter. [file elife-68558-supp1.pdf]

Supplementary File 1: Primer sets used for genotyping and RT-qPCR

Primers for genotyping

Gene	Forward Primer	Reverse Primer
Cc2d2a	TGTCCTGGAGCTCCCTTCTA	TGCAGGCAATTTCTCTGTTG (wt)
		CTTCACATCCATGCTGAGGA (mut)
Cre (generic)	ACATTTGGGCCAGCTAAACAT	CGGCATCAACGTTTTCTTTT
Islet1:cre	CATTTCACTGTGGACATTACTCCC	ATCTTCAGGTTCTGCGGGAA
mTmG	CTCTGCTGCCTCCTGGCTTCT	CGAGGCGGATCACAAGCAATA (wt)
		TCAATGGGCGGGGGTCGTT (gt)
Ptch1	GCCTGAAGAACGAGATCAGC (mut)	GTTTCCCAGCTTCCCTTTTC (mut)
	AGGGCTTCTCGTTGGCTACAA (wt)	CTGCGGCAAGTTTTTGGTTG (wt)
R26-EYFP	AAAGTCGCTCTGAGTTGTTAT	TAAGCCTGCCCAGAAGACTC (wt)
		GAAAGACCGCGAAGAGTTTG (mut)
Sox1:cre	GGCCCTCTCTTTGCGGTA	GGCAAACGGACAGAAGCATT
Tctn2	AGTGTGTCCTACTTAGGGCTTTTG (wt)	TCCTTCAGTGGTGAACCTCAAC (wt)
	CAGCAAGAACCATGTCTGGA (mut/cond)	CTTCAGTGGTGAACCTCAACG (mut)
		ACGGGTGTCCTACATCCAAG (cond)
Tmem231	TCTAGGGAAACCTGGAGAAAC	GCATGCAAGGACTTAACCACT (wt)
		AGCTAGCTTGCCAAACCTACA (mut)
Tmem67	GGGTGGGATTAGATAAATGCCTGCTCT	GGCTATGGGTAGAATATTGTTCCAG (wt)
		GGACCTGGCGATTTGACGTCCTCAG (mut)

wt = wild-type allele
mut = mutant/null allele
cond = conditional allele
gt = gene trap

Primer sets used for RT-qPCR

Gene	Forward Primer	Reverse Primer
Actb	TTCTTTGCAGCTCCTTCGTT	ATGGAGGGGAATACAGCCC
Gli1	GGTGCTGCCTATAGCCAGTGTCCTC	GTGCCAATCCGGTGGAGTCAGACCC
Hprt	TCCTCCTCAGACCGCTTTT	CATAACCTGGTTCATCATCGC
Ptch1	CTCCTCATATTTGGGGCCTT	AATTCTCGACTCACTCGTCCA
Shh	CCAATTACAACCCCGACATC	GGCCAAGGCATTTAACTTGT
Ubc	TCCAGAAAGAGTCCACCCTG	GACGTCCAAGGTGATGGTCT
